# Smokeless Tobacco Use and Religiousness

**DOI:** 10.3390/ijerph6010225

**Published:** 2009-01-12

**Authors:** Frank Gillum, Thomas O. Obisesan, Nicole C. Jarrett

**Affiliations:** 1 Howard University College of Medicine, 1112 Nora Drive, Silver Spring, MD 20904, USA E-mail: tobisesan@howard.edu; 2 W. Montague Cobb/NMA Health Institute, 1012 Tenth Street, NW, Washington, DC 20001, USA E-mail: njarrett@NMAnet.org

**Keywords:** Smokeless tobacco, religion, spirituality, smoking

## Abstract

Although smoking shows a strong negative association with religiousness, no studies have appeared of use of smokeless tobacco (ST) and religiousness. To assess an association of use of ST and religiousness, data from 9,374 men aged 17 years and over with complete data on self-reported frequency of attendance at religious services and use of smokeless tobacco were analyzed. Among men aged 17–29 years, 4.9% of frequent attenders (>=24 times/y) and 9.4% of others (<24 times/y) were current users of ST (p=0.002). After adjusting for multiple confounders by logistic regression, infrequent attenders were twice as likely as frequent attenders to be ST users: odds ratio 2.09, 95% confidence limits 1.12–3.92, p=0.02. This negative association suggests a protective effect of early-life religiousness on ST use, which might be taken into account in planning ST prevention efforts.

## 1. Introduction

Overall U.S. per capita consumption of tobacco products has declined steadily since 1955 [1]. Prevalence of cigarette smoking has declined among U.S. adults from 42.4% in 1965 to 21.5% in 2003 [2]. Some smokers may have switched to smokeless tobacco rather than ceasing to use any tobacco products [3]. However, smokeless tobacco has been associated with oral and pancreatic cancers, other oral pathology, and adverse pregnancy outcomes [4]. Further, ST use in youth may be a gateway for smoking initiation [5–7]. Analyses of prevalence data from the National Health Interview Surveys of 1987 to 2000 revealed low (< 1%) prevalence among women, and steadily declining prevalence from 6.1% to 4.5% (26%) among men (8, 9]. Analyses of data from the Current Population Survey showed similar trends through 2002 [10].

Compared to smoking, little is known about the social and cultural factors associated with smokeless tobacco use [11]. High prevalence has been documented in adolescent and young adult European American and American Indian males with low education levels living in the rural South and West [8]. A central component of culture, religion has been defined in many ways by a variety of scholars. In the American context, religiousness generally is characterized by belief in a higher power, identification with a religious tradition, affiliation with a religious institution, attendance at religious services, prayer and other religious behavior [12]. Surveys find these to be more prevalent in the U.S. than in any other industrialized nation and reports from analysis of population-based data have been published on an inverse association of smoking with religiousness in the U.S. population [12–14]. Yet no reports were found on use of smokeless tobacco and religiousness. In order to test the hypothesis that frequent attendance at religious services is associated with lower prevalence of smokeless tobacco use among adults of all ages independent of age, gender, ethnicity, education, region, and health status in the American population, data on a large, multi-ethnic sample of adults from the Third National Health and Nutrition Examination Survey (NHANES III) were examined.

## 2. Methods

The Third National Health and Nutrition Examination Survey (NHANES III) was conducted in 1988–1994 on a nationwide multi-stage probability sample of 39,695 persons from the civilian, non-institutionalized population aged two months and over of the United States excluding reservation lands of American Indians. Details of the survey plan, sample design, operations and response rates have been published as have procedures used to obtain informed consent and to maintain confidentiality of information obtained [15].

Demographic data including race category and ethnicity, years of education completed, medical history including self-assessed health status and behavioral information including frequency of attending church or religious services and smoking history were collected by household interview. Persons were asked, “How often do you attend church or religious services?” Values ranged from 0 to 1,825 times per year. Values in excess of 365 (n=7) were coded missing as well as four “don’t know” responses. For this analysis, persons reporting >=24 times per year are termed frequent attenders and those reporting 0–23 times per year are termed infrequent attenders. No information was available on other dimensions of religiousness in this survey.

Persons were asked “Have you ever used chewing tobacco or snuff?” Those responding “yes” were asked, “At what age did you first start using chewing tobacco or snuff fairly regularly?” “Do you use chewing tobacco or snuff now?” “Which—chewing tobacco or snuff?” “How many containers do you use per week?” Smoking status was determined by responses to the following questions: “Have you smoked at least 100 cigarettes during your entire life?” Those responding “yes” were asked, “How old were you when you first started smoking cigarettes fairly regularly?” and “Do you smoke cigarettes now?” Current smokers, who responded “yes” were asked, “About how many cigarettes do you smoke per day.” Persons reporting “less than 1 cigarette per day” were assigned a value of 0.5 cigarettes per day in this report, the minimum value; the maximum value was 140. Those responding “don’t know” or “varies/varied” were coded missing. Among 33,994 interviewed persons (85.6% of 39,695 sample persons), 13,932 were under age 17 and therefore excluded. Another 16 were excluded for missing ever-smoker status, 17 for missing data on ever using smokeless tobacco, and 35 for missing frequency of church attendance. Complete data were available for 19,994 persons including 9,374 men forming the present analysis sample.

The validity of self-reported ST use was confirmed by analysis of serum cotinine levels determined using an enzyme immunoassay method described elsewhere [13, 14]. Cotinine, (*S*)-1-methyl-5-(3-pyridinyl)-2-pyrrolidinone, is a major metabolite of nicotine with an *in-vivo* half life of 15–20 hours (compared to 0.5–3.0 hours for nicotine). It thus serves as a biomarker of exposure to tobacco. In self-reported never-smokers, mean serum cotinine (ng/mL) was 3.93 in self-reported never ST users, 11.70 in former ST users, and 328.80 in current ST users. Similarly in self-reported former smokers, mean serum cotinine was 17.06 in self-reported never ST users, 17.08 in former ST users, and 289.34 in current ST users. Validity of self-reported smoking has been reported previously [13, 14].

Statistical analysis: Detailed weighted descriptive statistics including percentiles and inter-quartile range (IQR) were computed using standard methods. Multivariate logistic regression analysis was used to control for confounding of the association of ST use with frequency of attendance at religious services. All models controlled for age in years. Regression analyses were performed using SUDAAN procedures, techniques that incorporated sampling weights and design features of the survey [16].

## 3. Results and Discussion

Among US men 17 and over, an estimated 5.1 million (5.74%, 95% CL 4.70%–7.00%) were current users of smokeless tobacco and 7.7 million (8.57%, 95% CL 7.47%–9.82%) were former users. Only 0.69% (0.47%–1.02%) of US women 17 and over reported current use and 0.55% (0.35%–0.87%) former use. Because of the small number of female users in the sample, the remaining analyses were done for men only. Among all ever ST users, the median age at which they started using regularly was 17 (IQR 14–24), with 90% starting by age 38. Only 5% had started by age 10. Of ever users 40.1% were still using ST at the time of interview. Among current ST users, 53% used chewing tobacco, 40% snuff, and 7% both; 28% were current cigarette smokers and 41% were former cigarette smokers. Chewing tobacco users used a median two (IQR 1–4) containers per week and snuff users used a median of three (IQR 1–7) containers per week.

Table 1 shows the prevalence of ST use by religious attendance stratified by selected demographic variables. Among men aged 17 and over, 4.4 % (95% CL 3.3–5.9%) of frequent attenders and 6.5% (95% CL 5.2–8.2) of others were current users of ST and 7.3% (5.5–9.5) and 9.3% (8.0–10.8%) were former users, respectively (p < 0.0001). The difference by attendance frequency was most pronounced in men aged 17–29 (p=0.002), less at aged 30–59 (p=0.23) and absent above age 60 (p=0.66). Further, it was seemed limited to European American men (p < 0.0001). Similar associations were seen in smokers and non-smokers, married and unmarried (Table 1).

Among ever regular users of ST, median age at first regular use of ST was 18 (IQR 14–30) among frequent attenders and 17 (IQR 14–23) among infrequent attenders (includes never attenders). Among current snuff users, frequent attenders used a median number of two (IQR 1–5) containers/week compared to infrequent attenders who smoked a median of three containers/week (IQR 1–7). Among current chewing tobacco users, frequent attenders used a median number of two (IQR 1–4) containers/week compared to infrequent attenders who also used a median of two containers/week (IQR 1–5).

The statistical significance of effect modification by age but not ethnicity was confirmed in logistic regression models with interaction terms for age (p=0.007) and ethnicity (p=0.15). Therefore further analyses were stratified by age (17–29, 30–59, 60+). Models were fit within age strata, with ST status (current user versus non-user) as the dependent variable and frequent-attender status (yes, no) as the exposure variable in men adjusting for age or age and other confounders. Table 2 shows odds ratios (OR) and 95% confidence limits within strata. At age 17–29, infrequent attenders were about twice as likely to be ST users even after controlling for age (p=0.01), or age, ethnicity, region, education, and health status (p=0.02). The odds ratio was little changed when the analysis was restricted to nonsmokers. No significant association was seen over age 30.

Cigar and pipe smoking were also examined. Among ever smokers the percent currently smoking fell from 20% at age 17–29 to 12% at age 60+ for cigars and from 13% to 8% for pipes. Adjusting for multiple confounders, ever smoking cigars was weakly associated with attendance at age 17–29 (OR=1.71, 95% CL 0.99–2.95, P=0.05), but not 30–59 (OR=1.22, 95% CL 0.96–1.56, p=0.09), or 60+ (OR=1.21, 95% CI 0.96–1.51, p=0.10). No significant associations were seen for ever smoking a pipe. Analyses of attendance and current smoking were not done because of small numbers within some strata.

In this national sample of American men aged 17 and over, ST use was much less prevalent among frequent attenders of religious services than among infrequent or never attenders independent of age, ethnicity, gender and other confounders. In this first report on ST use and religiousness, the *a priori* hypothesis was confirmed consistent with a possible beneficial effect of greater religiousness on ST use in young men. This negative association suggests a protective effect of early-life religiousness on ST use, which should be taken into account in planning ST prevention efforts and in analysis of data on association of ST use with other variables such as ethnicity.

Possible mechanisms by which high frequency of attendance at religious services may be associated with low prevalence of ST use may derive from the correlation of this single behavior with other dimensions (e.g. intrinsic and extrinsic) of religiousness. High religiousness may reduce prevalence of tobacco use by simple direct effects such as the prohibition of tobacco use among Seventh Day Adventists and Mormons and the more recent phenomenon of church-sponsored health promotion activities including anti-smoking messages for members and surrounding communities [12]. Because of its association with other disreputable behavior such as excessive drinking and gambling, many Protestant Christian denominations strongly discouraged tobacco use prior to the 1940’s, especially among women and youths [17]. After World War II tobacco use became more widely acceptable. However, as public recognition of the health hazards of smoking grew since the 1964 Surgeon General’s Report, leaders and members of religious institutions shared in that recognition. Today, smoking or chewing tobacco is not countenanced in many religious buildings or functions as in many other public places. Unhealthful habits such as use of tobacco and other drugs are generally discouraged in religious teaching, because they do not honor the body as the temple of the spirit [12]. The lack of association of religious attendance with pipe smoking may relate to its more respectable image in western culture.

Indirect mechanisms for an effect of religiousness on tobacco use include religion’s ability to reduce the emotional impact of stressful life situations, prevent depression and enhance coping, thus reducing the need to use tobacco or other drugs to relieve the effects of stress [12, 17]. Religious communities provide social support for healthy behaviors such as tobacco avoidance. Available evidence suggests that religiousness reduces smoking prevalence by reducing initiation among adolescents and young adults [18, 19]. Similar mechanisms may operate for ST use. However, in cross-sectional, observational studies, non-causal mechanisms for the observed association of frequency of attendance and smoking must also be considered, e.g. non-users may feel more comfortable than users at religious services and hence are more likely to attend.

Limitations of the present study include possible bias arising from survey non-response and from missing values for some variables. Special studies of NHANES data have indicated little bias due to non-response [15]. At least 12 dimensions of religiousness have been defined [12]. Attendance at religious services is an indicator of organizational religiousness. Since data on multiple dimensions were unavailable in NHANES III, it was used because it is correlated with other dimensions of public and private religiousness and provided data that are directly comparable with a body of research data on this variable spanning many decades. Moderate over-reporting of religious attendance is likely [12]; however the NHANES III variable should serve well to separate frequent from infrequent attenders. Adequate reliability has been documented for self-reported smoking in a number of populations [13]. Whether this extends to ST use is unknown. Confounding by variables not controlled for cannot be excluded. The representativeness of the sample and the use of sample weights provides generalizability of the results to United States non-institutionalized population of the same ages. Future research should include large surveys of multiple dimensions of religiousness/spirituality and longitudinal studies of ST initiation, duration, and cessation in population-based samples of men to determine temporal sequence of the relationship.

## 4. Conclusions

In conclusion, greater frequency of attendance at religious services was associated with lower prevalence of ST use in men aged 17–29 years in a national, multi-ethnic sample. This association was weaker or absent above age 30. Similar to previous findings for smoking, exposure to religious beliefs and activities in childhood and adolescence may prevent or delay adoption of ST use. Future research should examine several dimensions of religiousness in adolescents and young adults as predictors of subsequent ST and other tobacco use in longitudinal studies.

## Figures and Tables

**Table 1.** Prevalence (percent) of self-reported tobacco use by attendance at religious services among 9,374 men. CI, confidence interval; EA, Non-Hispanic European American; AA, Non-Hispanic African American; MA, Mexican American. Never users not shown.

	Attendance (times/y) and smokeless tobacco use status			
	0–23		24+	
	Current	Former	Current	Former
**N**	282	411	156	255
**All**	6.5	9.3	4.4	7.3
**17–29**	9.4	11.7	4.9	7.1
**30–59 y**	4.9	7.9	3.6	6.9
**60+y**	6.1	9.2	5.8	8.2
**EA**	7.8	10.8	5.2	8.6
**AA**	3.1	5.0	3.0	5.0
**MA**	1.7	3.7	1.3	4.0
**Smoker**	5.4	10.9	4.1	7.0
**Fmr smoker**	9.2	11.7	6.0	11.4
**Nvr smoker**	5.7	5.7	3.3	4.1
**Married**	7.0	10.5	4.1	8.6
**Unmarried**	5.9	7.8	5.0	4.2
**Metro**	2.9	7.4	1.6	4.6
**Nonmetro**	10.6	11.5	6.8	9.5

**Table 2.** Adjusted odds ratios (95% CI) of current use of smokeless tobacco in infrequent attenders compared with frequent attenders of religious services by age in men. OR, odds ratio; CI, confidence interval;*adjusted for age, African American ethnicity, Mexican American ethnicity, education < 12 years, region (South vs other), fair/poor self-reported health + p<=0.01, ** p<=0.05.

Age (y)	N	Age-adjusted OR	95% CI	Confounder*-adjusted OR	95% CI
17–29	2385	2.14	1.18–3.87+	2.09	1.12–3.92**
30–59	3885	1.41	0.78–2.54	1.41	0.79–2.52
60+	3104	1.07	0.74–1.54	1.04	0.71–1.52
